# Mandibular fractures in aged patients – Challenges in diagnosis

**DOI:** 10.1111/edt.12778

**Published:** 2022-08-11

**Authors:** Leena Kannari, Emilia Marttila, Hanna Thorén, Miika Toivari, Johanna Snäll

**Affiliations:** ^1^ Department of Oral and Maxillofacial Diseases University of Helsinki and Helsinki University Hospital Helsinki Finland; ^2^ Department of Oral and Maxillofacial Surgery, Institute of Dentistry University of Turku Turku Finland; ^3^ Department of Oral and Maxillofacial Diseases Turku University Hospital Turku Finland

**Keywords:** geriatric patient, aged patient, mandibular fracture, missed diagnose

## Abstract

**Background/Aims:**

Delayed treatment of a mandibular fracture can lead to complications. Therefore, early diagnosis is important. The aim of this study was to clarify the specific features of mandibular fractures in aged patients and the effect of age on possible missed diagnoses.

**Material and Methods:**

Patients aged over 60 years with a recent mandibular fracture were included in the study. The outcome variable was a missed mandibular fracture during the patient's first assessment in the primary health care facility. Predictor variables were age group, categorized as older adults (aged ≥60 and <80 years), elders (aged >80 years), patient's age as a continuous variable and age sub‐group divided into decades. Additional predictor variables were the patient's memory disease and injury associated with intracranial injury. Explanatory variables were gender, injury mechanism, type of mandibular facture, combined other facial fracture, edentulous mandible/maxilla/both, surgical treatment of the mandibular fracture, and scene of injury.

**Results:**

Mandibular fractures were missed in 20.0% of the 135 patients during their first healthcare assessment. Significant associations between missed fractures and age group, gender, fracture type, or injury mechanism were not found. By contrast, memory disorder (*p* = .02) and site of injury (*p* = .02) were significantly associated with missed fractures. Fractures were missed more frequently in patients who were in hospital or in a nursing home at the time of injury.

**Conclusions:**

There is an increased risk of undiagnosed mandibular fractures in the aged population. Small injury force accidents may cause fractures in old and fragile individuals. Careful examination is necessary, especially in patients with memory disorder.

## 1 INTRODUCTION

In patients aged over 60 years, the most common injury mechanism leading to a mandibular fracture is a ground‐level fall.^1^, ^2^ The most common fracture site is the condyle.^1^, ^2^ Although the majority of these fractures can be treated without surgery,^2^, ^3^, ^4^ oral and maxillofacial evaluation, possible immobilization and patient guidance with appropriate follow up are needed. The choice of optimal treatment must take into account the patient's general health, nutrition, dental condition, type of fracture, and the effect of the treatment modality on the quality of life of the patient.

With age, the risk of several diseases, such as osteoporosis, cardiovascular diseases, memory disorders, and cerebrovascular disorders, increases.^5^, ^6^, ^7^, ^8^, ^9^ In addition, balance, muscle strength, and posture control deteriorate and reflexes slow down in older people.^10^, ^11^ Memory disorders increase the risk of falling by each point of lowered MINI‐MENTAL State Examination (MMSE) results from 30 to 22 with a rate ratio of 1:20.^12^ Bones may become brittle^13^ and teeth may be lost, leading to lowered alveolar bone volume in the mandible.^14^ Age‐induced cognitive impairment^15^ and memory disorders can lead to difficulties in reporting the injury event or symptoms, causing diagnostic difficulties. Delayed treatment can result in complications such as infection, prolonged pain, numbness, malocclusion,^16^, ^17^ and malnutrion, thus potentially causing a collapse in the patient's general health.^18^

Previous studies have shown that mandibular fractures can be challenging to diagnose especially in young children,^19^ while the number of facial fractures increases in older age groups.^20^ The aim of this study was to clarify the specific features of mandibular fractures in patients aged over 60 years. A particular aim was to clarify the possible effect of aging on the diagnostic accuracy of mandibular fractures. The hypothesis was that predisposing factors for missed fractures can be found.

## 2 MATERIALS AND METHODS

The internal review board of the Head and Neck Center of Helsinki University Hospital approved the study protocol (HUS/356/2017).

The records of all patients over 60 years diagnosed with a recent mandibular fracture at the Emergency Unit of Oral and Maxillofacial Surgery at Helsinki University Hospital between January 1, 2013 and December 31, 2018 were included. All injury‐related patient records were retrieved retrospectively from electronic patient records.

The outcome variable was a missed mandibular fracture. A missed fracture was determined when a fracture was not suspected or diagnosed during the patient's first primary healthcare assessment.

The primary predictor variable was the age group, categorized as older adults (aged ≥60 and <80 years) and elders (aged >80 years). The secondary predictor variable was the patient's age as a continuous variable and the age sub‐group divided into decades. Additional predictor variables were the patient's memory disease and any injury associated with an intracranial injury.

Explanatory variables were gender, injury mechanism, type of mandibular facture, combined other facial fracture, edentulous mandible/maxilla/both, surgical treatment of the mandibular fracture, and scene of injury. Injury mechanisms were grouped into the following 6 categories: (1) assault, (2) ground‐level fall, (3) bicycle accident, (4) traffic accident, (5) fall from height, and (6) other. Fractures of the mandible were further classified as: (1) isolated unilateral fractures of the mandibular condyle, ramus, or both, (2) isolated mandibular fracture in the tooth‐bearing region, and (3) multiple mandibular fractures. The scene of the injury was defined as hospital, nursing home, home or other.

The other variables were clinical symptoms and findings categorized as skin wounds and contusions, mucosal wounds, pain, swelling of the face, bruise in the facial area, change in occlusion, neurosensory disturbance, restricted mouth opening, dental injury, and bleeding from the ear. Associations between clinical symptoms and findings and missed diagnoses were evaluated. In addition, the number of days from injury to fracture diagnosis and the association between delayed assessment and missed diagnosis were reported.

Data were analyzed using GraphPad Prism version 5.00 (GraphPad Inc.). A two‐tailed Mann–Whitney test was used to assess the significance of differences in continuous variables. Fisher's exact test was applied to examine the association between variables with nominal scales. *p* values of less than .05 were considered significant.

## 3 RESULTS

A total of 135 patients with mandibular fractures were included in the study. The age of the patients ranged from 60.6 to 94.2 years (mean 73.7 years, median 72.9 years). In all, 76.3% (103) were older adults and the remaining 32 patients (24.7%) were elders (Table 1). Just over half were women (56.3%). The most common injury mechanism was a ground‐level fall (82.2%), followed by a bicycle accident (5.2%) and traffic accident (3.7%). The most common mandibular fracture type was an isolated unilateral mandibular condyle or ramus fracture (57.8%), and most of the fractures were not associated with other fractures of the facial area (83.0%; Table 2).

**TABLE 1:** Age variation of 135 mandibular fracture patients aged over 60 years

Age (years)	
Range	60.6–94.2
Mean	73.7
Median	72.9

	No. of patients	%
	(*n* = 135)	
Age group (years)		
≥60 and <80	103	76.3
≥80	32	23.7
Age (years)		
60–69	54	40.0
70–79	49	36.3
80–89	22	16.3
≥90	10	7.4

**TABLE 2:** Descriptive statistics for 135 patients with mandibular fracture

	No. of patients	%	No. of patients in age group ≥60 and <80 years		No. of patients in age group ≥80 years		*p*
			*n*	%	*n*	%	
Gender							
Male	59	43.7	49	83.	10	16.9	*.15*
Female	76	56.3	54	71.1	22	28.9	
Injury mechanism							
Ground‐level fall	111	82.2	81	73.0	30	27.0	*.2*
Bicycle accident	7	5.2	7	100.0	0	0.0	
Traffic accident	5	3.7	3	60.0	2	40.0	
Assault	4	3.0	4	100.0	0	0.0	
Fall from height	4	3.0	4	100.0	0	0.0	
Other	4	3.0	4	100.0	0	0.0	
Mandibular fracture							
Multiple mandibular fracture	49	36.3	34	69.4	15	30.6	*.34*
Isolated unilateral mandibular condyle‐ramus fracture	78	57.8	63	80.8	15	19.2	
Isolated mandibular tooth‐bearing region fracture	8	5.9	6	75.0	2	25.0	
Combined other fracture							
Yes	23	17.0	15	65.2	8	34.8	*0.18*
No	112	83.0	88	78.6	24	21.4	
Mandibular fracture type							
Condylar	117	86.7	90	76.9	27	23.1	*0.5*
Symphysis/parasymphysis	22	16.3	18	81.8	4	18.2	
Corpus	15	11.1	10	66.7	5	33.3	
Ramus and/or coronoideus	7	5.2	7	100.0	0	0.0	
Angle	5	3.7	4	80.0	1	20.0	
Edentulous mandible/maxilla/both							
Yes	18	13.3	8	44.4	10	55.6	***0.0017***
No	117	86.7	95	81.2	22	18.8	
Surgical treatment							
Yes	36	26.7	29	80.6	7	19.4	*0.65*
No	99	73.3	74	74.7	25	25.3	
Memory disorder							
Yes	13	9.6	7	53.8	6	46.2	*0.079*
No	122	90.4	96	78.7	26	21.3	
Scene of injury							
Hospital/Nursing home	11	8.1	3	27.3	8	72.7	***0.0344***
Home	32	23.7	24	75.0	8	25.0	
Other	92	68.15	76	82.6	16	17.4	
Traumatic brain injury							
Yes	7	5.19	5	71.4	2	28.6	*0.67*
No	128	94.81	98	76.6	30	23.4	

Bold italics *p*‐values are statistically significant.

The associations between study variables and age groups are presented in Table 2. Fractures of 27 patients (20.0%) were missed at the first healthcare examination. In these patients, the fracture was diagnosed a mean of 11 days after the injury, whereas in patients without a missed fracture, the diagnosis was made on the day of injury (Table 3).

**TABLE 3:** Associations between predictors and diagnosis delay in 135 patients with mandibular fracture

	Patients with missed diagnosis	Patients without missed diagnosis
Days from injury to fracture diagnosis		
Mean	11.3	1.2
Median	3	0
Range	1–57	0–45

	*n*	%	*n*	%	*p*
All	27	20.0	108	80.0	
Age (years)					
Mean	73.1		73.9		
Range	60.6–91.3		60.7–94.2		
Age group					
≥60 and <80	21	20.4	82	79.6	*1.0000*
≥80	6	18.8	26	81.3	
Age (years)					
60–69	12	22.2	42	77.8	*0.9612*
70–79	9	18.4	40	81.6	
80–89	4	18.2	18	81.8	
≥90	2	20.0	8	80.0	
Gender					
Male	14	23.7	45	76.3	*0.3891*
Female	13	17.1	63	82.9	
Injury mechanism					
Ground‐level fall	23	20.7	88	79.3	*0.7411*
Bicycle accident	2	28.6	5	71.4	
Traffic accident	0	0.0	5	100.0	
Assault	1	25.0	3	75.0	
Fall from height	0	0.0	4	100.0	
Other	1	25.0	3	75.0	
Mandibular fracture					
Multiple mandibular fracture	9	18.4	40	81.6	*0.4401*
Isolated unilateral condyle‐ramus fracture	15	19.2	63	80.8	
Isolated tooth‐bearing region fracture	3	37.5	5	62.5	
Combined other facial fracture					
Yes	3	13.0	20	87.0	*0.5671*
No	24	21.4	88	78.6	
Mandibular fracture type					
Condylar	24	20.5	93	79.5	*0.5705*
Symphysis/parasymphysis	6	27.3	16	72.7	
Corpus	2	13.3	13	86.7	
Ramus and/or coronoideus	0	0.0	7	100.0	
Angle	1	20.0	4	80.0	
Edentulous mandible/maxilla/both					
Yes	4	22.2	14	77.8	*0.758*
No	23	19.7	94	80.3	
Surgical treatment					
Yes	5	13.9	31	86.1	*0.3389*
No	22	22.2	77	77.8	
Memory disorder					
Yes	6	46.2	7	53.9	***0.0234***
No	21	17.2	101	83.0	
Scene of injury					
Hospital/Nursing home	5	45.5	6	54.6	***0.0175***
Home	3	9.4	29	90.6	
Other	19	20.7	73	79.4	
Traumatic brain injury					
Yes	1	14.3	6	85.7	*1.0000*
No	26	20.3	102	79.7	

Bold italics *p*‐values are statistically significant.

Fractures were missed most often in patients aged 60–70 years (22.2%) and over 90 years (20.0%). However, there were no significant differences between older adults and elders or between age sub‐groups (Table 3). No significant associations with diagnosis delay were observed for injury mechanisms or fracture types. However, a significant association was noted between missed fractures and memory disorder (*p* = .02). Fractures were missed in 46.2% of patients with memory disorder compared with 17.2% of patients without a diagnosed memory disorder.

Interestingly, 45.4% of patients with a missed diagnosis were admitted to hospital or lived in a nursing home at the time of injury. By comparison, 90% of patients with a correct diagnosis at the first healthcare contact lived at home (*p* = .03; Table 2).

An average of 3.6 different symptoms or clinical findings were observed in the patients. The most common clinical finding or symptom was pain, followed by a skin wound on the lower face and restricted mouth opening (Table 4). No significant differences were present for symptoms or clinical findings in patients with missed fractures and patients whose fracture was suspected/diagnosed at the first healthcare contact.

**TABLE 4:** Associations between clinical findings and symptoms in 135 patients with mandibular fracture

	Patients with missed diagnosis		Patients without missed diagnosis		*p*
	*n*	% of 27 patients	*n*	% of 108 patients	
Pain	22	81.5	84	77.8	*0.7972*
Skin wound on lower face	16	59.3	64	59.3	*1*
Restricted mouth opening	12	44.4	46	42.6	*1*
Change in occlusion	10	37.0	33	30.6	*0.6446*
Dental injury	7	25.9	35	32.4	*0.644*
Swelling	7	25.9	36	33.3	*0.4997*
Facial bruises	7	25.9	41	38.0	*0.2704*
Skin contusion on lower face	5	18.5	21	19.4	*1*
Mucosal wound	4	14.8	21	19.4	*0.783*
Bleeding from ear canal	2	7.4	10	9.3	*1*
Neurosensory disturbance	1	3.7	2	1.9	*0.4909*

Italicized values are *p*‐values.

## 4 DISCUSSION

This study evaluated the specific features of mandibular fractures in elderly patients aged 60 years or more. The specific aim was to clarify the possible effect of age on missed diagnosis. The hypothesis was that predisposing factors for missed fractures would be found. This hypothesis was confirmed, as 20.0% of the mandibular fractures were not diagnosed at the first healthcare contact. Significant predictive factors for missed fractures were the patient's memory disorder (*p* = .02) and the scene of injury (*p* = .03). The fracture was most likely missed if the patient was in hospital or at a nursing home at the time of the accident. In addition, missed fractures led to a significant delay in diagnosis (mean 11.3 days, median 3 days).

A ground‐level fall has been reported to be the most common cause for facial fractures in elderly persons.^1^, ^21^, ^22^ This study is in line with previous research since in 4 out of 5 patients, the injury mechanism was a ground‐level fall. The literature has shown that the rate of underdiagnosis in geriatric trauma can reach up to 69.1% in patients aged at least 70 years,^23^ as low‐energy injuries do not necessarily raise a suspicion of injuries. Comprehensive examination of the face for signs of facial fractures may be omitted. Special characteristics of the aging population and careful assessment, including facial injuries, should be emphasized in emergency care processes in elderly patients.

Memory disorder proved to be a significant predictor of missed mandibular fractures. Diagnosing fractures in patients with memory disorder may be challenging since the patient might be unable to report symptoms appropriately. It is also possible that the accident had no eye‐witnesses, and the patient is unable to report the injury at all. The prevalence of previously unregistered cognitive impairment in elderly emergency patients is high,^24^ but dementia often remains undetected.^25^ In addition, multiple morbities are common in these patients and they also have deficiency of functions such as impaired hearing and vision,^25^ which can complicate patient examination and diagnosis. These emphasize the role of health care professionals in the detection of memory disease during emergency visits and the particularly careful diagnosis of fractures in these patients. This study revealed that the clinical symptoms and findings of fractures were evident with the most common of these being pain, a wound on the lower face, and restricted mouth opening. It is essential to train healthcare professionals who work with memory disease patients, and with elders in general, regarding facial fractures, especially in view of the aging population worldwide.

Injuries in a hospital or at a nursing home were significantly associated with a missed diagnosis. This highlights the importance of training nursing staff regarding facial fractures. Patients in these facilities are likely to have many other treatment needs, and thus, the facial area may receive less attention. In addition, ground‐level falls occur often in nursing homes,^24^ and staff may become accustomed to fall injuries and consider them to be habitual and minor.

With age, the risk of accidents increases due to age‐related changes in balance, muscle strength, posture control, reflexes, and many other common diseases.^10^, ^11^, ^13^ In the mandibular region, tooth loss may lead to lowered alveolar bone volume.^14^ The atrophic mandible is thin and vulnerable to fractures.^14^ As shown in an earlier study, tooth loss increases with age.^26^ It is also common in nursing home residents.^27^, ^28^, ^29^ However, tooth loss did not explain the diagnostic challenges in this study.

**FIGURE 1:**
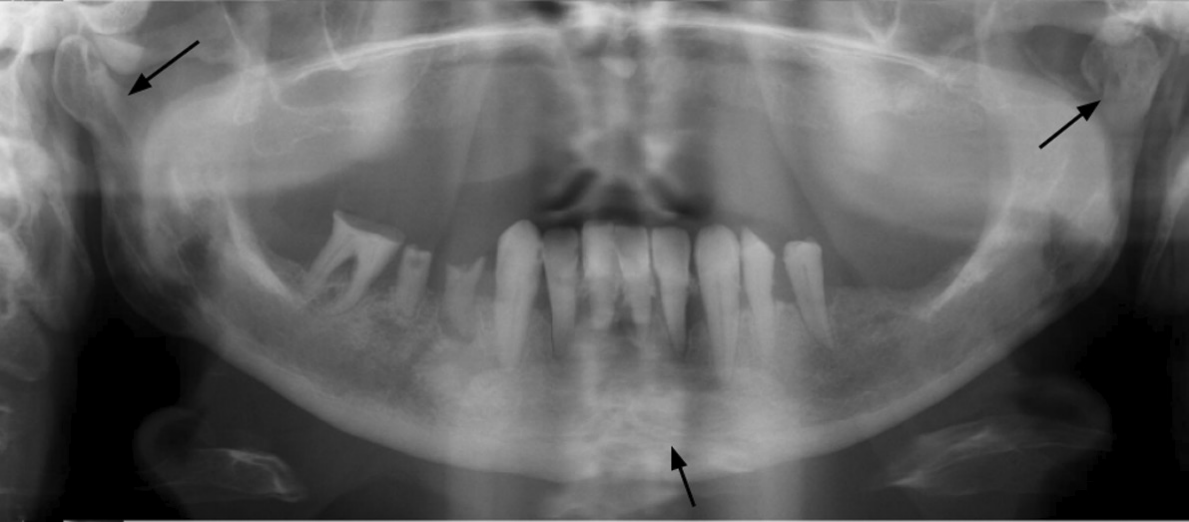
A 63‐year‐old woman with no diagnosed diseases or medications visited emergency care the same day after falling on the ground. The patient was examined at the hospital emergency polyclinic, and the skin wound on the anterior lower jaw was sutured. Despite pain in the jaw, the patient did not seek further emergency services until 2 weeks later when the skin had become red and swollen, and the edges of the wound were infected. During the second evaluation, a sub‐mental abscess was detected. The patient was referred to an ear, nose, and throat clinic, from which she was referred onward to maxillofacial surgery care. Clinical examination raised suspicion of a mandibular fracture. Dental panoramic tomography showed bilateral mandibular condyle fractures and suspicion of a symphyseal fracture (arrows).

**FIGURE 2:**
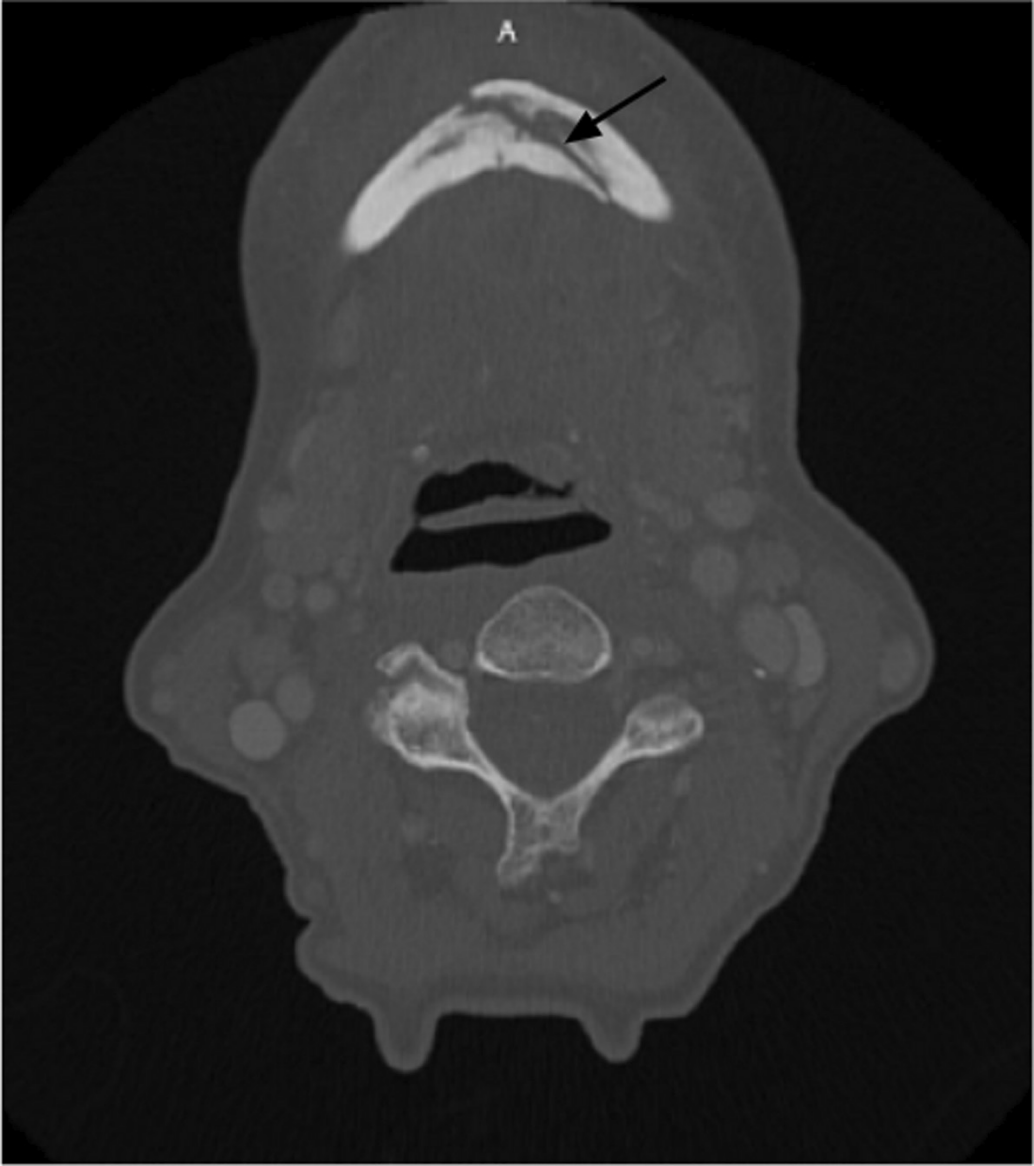
Computer tomography confirmed the diagnosis of a symphyseal fracture (arrow)

**FIGURE 3:**
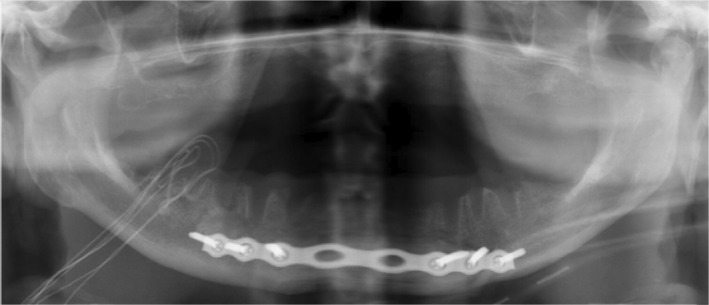
The patient underwent surgery for the sub‐mental abscess and the fractures. The symphysis fracture was repositioned and fixated with titanium plates and screws, and residual teeth were removed 14 days after the injury.

Overall, only 26.7% of patients in this study underwent surgical treatment for the fracture. The high number of condylar fractures combined with tooth loss may explain the low need for surgery. Patients over the age of 80 years underwent surgical treatment slightly less frequently than patients in the younger age group, but the difference was not statistically significant. Also missed fractures were treated with surgery slightly less often (18.5%) than fractures without a missed diagnosis (28.7%). However, it should be noted that more than one‐fifth (22.7%) of the missed fractures were eventually surgically treated. Additionally, a missed fracture led to a significant delay in diagnosis. Although studies have not shown a clear association between delayed surgical treatment and complications of mandibular fractures,^30^, ^31^ early diagnosis is clinically relevant (Figures 1, 2, 3). In fragile elders, mandibular fractures may lead to nutritional challenges due to pain and chewing problems. These may in turn lead to a decline in overall health.^32^, ^33^ All in all, early diagnosis of mandibular fractures guarantees an optimal surgical schedule, and the patient can be given instructions for proper pain management and an appropriate diet.

Surgical treatment of mandibular fractures in the elderly requires careful planning, taking into account possible complications and the patient's eligibility for anesthesia as well as the prognosis for fracture recovery. Among other things, the patient's disease and medication history should be carefully considered. For example, bisphosphonate therapy can predispose to bone healing problems^34^ and osteoporosis can predispose to bone healing delays.^35^ However, age should not be an exclusion criterion for any treatment. Instead, it is important to evaluate treatment options individually to maintain quality of life in these aged patients.

Limitations of this study include its retrospective nature and the relatively small number of patients. A retrospective study entails the risk of patient records containing incomplete entries of symptoms, clinical findings, and location of the injury. In addition, data from patients with completely missed fractures could not be included in the study.

The findings highlight the need for continuing education and facial fracture awareness among healthcare professionals. Despite clinically significant signs, up to one‐fifth of fractures were missed at the first healthcare contact. To maintain optimal treatment for aged patients, medical doctors, dentists, and other health professionals should be aware of the importance of careful clinical examination, and they should know the typical mandibular fracture symptoms and findings.

## AUTHOR CONTRIBUTIONS

Leena Kannari, DDS: Data collection, analysis, manuscript preparation. Emilia Marttila, MD, DDS, PhD: Statistical analysis, manuscript preparation. Hanna Thorén, MD, DDS, PhD, Professor: Study design, critical manuscript evaluation. Miika Toivari, MD, DDS, PhD: Study design, critical manuscript evaluation. Johanna Snäll, MD, DDS, PhD, Associate Professor: Study design, data collection, analysis, manuscript preparation.

## Funding information

No funding was received for this study.

## CONFLICT OF INTEREST

The authors declare that they have no conflict of interest.

## Data Availability

The data that support the findings of this study are available on request from the corresponding author. The data are not publicly available due to privacy or ethical restrictions.
